# Cost-effectiveness of diagnostic tests for threatened preterm labor in singleton pregnancy in France

**DOI:** 10.1186/s12962-018-0106-y

**Published:** 2018-06-14

**Authors:** Thomas Desplanches, Catherine Lejeune, Jonathan Cottenet, Paul Sagot, Catherine Quantin

**Affiliations:** 1 Service de Gynécologie-Obstétrique, Médecine Fœtale et Stérilité Conjugale, CHU de Dijon, 21000 Dijon, France; 2 EPICAD LNC-UMR1231, Burgundy & Franche Comte University, Dijon, France; 3 Clinical Epidemiology Unit, Inserm, CIC 1432, Dijon, France; 4 Clinical Epidemiology Unit, Clinical Investigation Center, Dijon University Hospital, Dijon, France; 5 Biostatistics and Bioinformatics (DIM), University Hospital, Dijon, France; 6 Bourgogne Franche-Comté University, Dijon, France; 7 Biostatistics, Biomathematics, Pharmacoepidemiology and Infectious Diseases (B2PHI), INSERM, UVSQ, Institut Pasteur, Université Paris-Saclay, Paris, France

**Keywords:** Cost-effectiveness, Diagnostic test, Economic evaluation, Preterm birth, Threatened preterm labor

## Abstract

**Background:**

Previous studies have showed that the early diagnosis of threatened preterm labor decreases neonatal morbidity and mortality, avoids maternal morbidity induced by antepartum bed rest and unnecessary treatment, and reduces costs. Although there are many diagnostic tests, none is clearly recommended by international guidelines. The aim of our study was to compare seven diagnostic methods in terms of effectiveness and cost using a decision analysis model in singleton pregnancy presenting threatened preterm labor, between 24 and 34 weeks of gestation.

**Methods:**

Seven diagnostic strategies based on individual or combined use of the following tests: cervical length, cervical fibronectin test, cervical interleukin test and protein in maternal serum, were compared using a decision analysis model. Effectiveness was expressed in terms of serious adverse neonatal events avoided (neonatal morbidity and mortality) at the hospital discharge. The economic analysis was performed from the health care system perspective. Deterministic and probabilistic analyses were performed to test the robustness of the model.

**Results:**

At 24–34 weeks of gestation, the association of cervical length and qualitative fibronectin was the most efficient strategy dominating all alternatives, reducing the perinatal death or severe neonatal morbidity rate up to 15% and the costs up to 31% according to the gestational age. This result was confirmed by the deterministic sensitivity analyses. The probabilistic analysis showed that the association of cervical length and qualitative fibronectin dominated cervical length < 15 mm in more than 90% of the simulations. The comparison with the other tests revealed more uncertainty.

**Conclusions:**

A test using cervical length and qualitative fetal fibronectin appears to be the best diagnostic strategy. Decisions regarding its generalization and funding in France in this population of women should take into account the high, lifetime costs induced by prematurity.

**Electronic supplementary material:**

The online version of this article (10.1186/s12962-018-0106-y) contains supplementary material, which is available to authorized users.

## Background

The main consequence of threatened preterm labor (TPL) is preterm birth, which is the leading cause of neonatal mortality and severe morbidity. Preterm birth is defined as birth before 37 weeks of gestation, but it is generally stratified in three groups according to the gestational age; 24–27 (extremely preterm), 28–31 (very preterm) and 32–34 (moderate preterm) weeks [1].

In developed countries, spontaneous preterm birth occurs in 6–13% of pregnancies [1]. TPL is considered as the cause of preterm birth in 45% of cases, the other causes being premature preterm rupture of the membranes in 25% and maternal or fetal infections in 30% of the cases [2]. TPL is also one of the main causes of hospitalization during pregnancy, and leads to substantial costs estimated at $820 million in the United States of America [3]. Treatment consists of prolonging pregnancy with tocolysis, reducing neonatal mortality and morbidities in cases of preterm delivery by injecting corticosteroids, and sometimes transfer to a specialized center [4]. Studies show that 75–95% of women with threatened preterm labor do not deliver within 7 days, and 40% will even deliver at term [5, 6]. Furthermore, 44% of these women have at least two subsequent admissions for preterm labor, thus leading to additional costs [6]. Therefore, it appears important to identify true TPL early in order to decrease neonatal morbidity and mortality, avoid maternal morbidity induced by antepartum bed rest [7] and unnecessary treatment, and to reduce costs.

According to the literature, diagnostic tests such as cervical length measurement, qualitative cervicovaginal fetal fibronectin (fFN), and cervicovaginal interleukin-6 (IL-6) have been proven to increase accuracy when predicting premature birth [8–14]. However, no diagnostic strategy is clearly recommended by international guidelines [15–17]. Cervical length measurement has proven to be a more efficient strategy than medical examination for predicting preterm birth in symptomatic women [14, 17], but currently neither cervical length measurement nor qualitative cervicovaginal fFN can be recommended, and further investigation is required [18].

These diagnostic methods have been assessed in several medico-economic analyses based on decision analysis models, whose results showed they could be accurate enough to be cost-effective [19–23]. However, newer diagnostic tests, such as quantitative fetal fibronectin or proteins in maternal serum, which have also shown significant results, were not included in previous cost-effectiveness studies [24, 25]. Subsequent antepartum hospitalizations were not taken into account either. Given the large number of strategies to consider and the lack of consensus regarding the optimal strategy to recommend, the objective of this study was to compare seven diagnostic methods in terms of cost and effectiveness using a decision analysis model in singleton pregnancy presenting threatened preterm labor.

## Methods

### Study population

A Medline literature research was performed. It was restricted to studies written in English or in French from 2004 onwards, and included “preterm labor, cervical length, fetal fibronectin, preterm birth” as key words. Most of the published studies considered (1) women with a singleton pregnancy, (2) hospitalized for TPL between 24 and 34 weeks gestational age with symptoms indicating threatened preterm delivery based on the presence of regular uterine contractions and intact membranes with possible cervical change but without advanced cervical dilation (< 3 cm), (3) with a preterm delivery (PTD) occurring within 7 days of the initial hospitalization, and (4) without severe maternal disease such as severe gestational arterial hypertension, pre-eclampsia, eclampsia, premature preterm rupture of the membranes, and placenta previa, or cases of termination of pregnancy for maternal and fetal medical reasons.

Only the studies using these inclusion criteria were taken into account in our study.

### General description of the model

A cost-effectiveness analysis was conducted using a decision analysis model. Seven diagnostic tests among women presenting with TPL were compared until hospitalization discharge. The choice between the seven tests was represented by a decision node. All clinical events in each strategy were then associated with estimated conditional transition probabilities. At the end of each alternative strategy of the decision tree, two payoffs were assigned corresponding to the total cost of care and the effectiveness. The decision tree was built and analyzed using TreeAge Pro 2017 software (TreeAge Software, Inc., Williamstown, MA).

### Description of strategies

Cervical length (CL) measured by transvaginal ultrasonography defined as positive if CL < 25 mm was considered as the reference strategy (S_ref_) because it appears to be the strategy the most widely used by French health care providers.

Other alternatives were:

S_2_: a qualitative rapid fetal fibronectin (fFN) test, defined as positive if fFN ≥ 0.05 µg/ml;
S_3_: a quantitative fetal fibronectin test, defined as positive if fFN ≥ 200 ng/ml;
S_4_: a cervical interleukin-6 test (IL-6), defined as positive if IL-6 ≥ 210 pg/ml;
S_5_: a combination test associating CL measured by transvaginal ultrasonography, plasma on activation normal T-expressed and secreted regulated (RANTES) and plasma interleukin-10, defined as positive if CL ≤ 18 mm, plasma RANTES ≥ 49 293 pg/ml and plasma interleukin-10 ≥ 48 pg/ml;
S_6_: CL measured by transvaginal ultrasonography, defined as positive if CL < 15 mm;
S_7_: a test associating CL and qualitative fetal fFN, defined as positive if CL < 15 mm or if CL is 16–30 mm and qualitative fetal fFN ≥ 0.05 µg/ml.

### Description of the decision tree

**Fig. 1:**
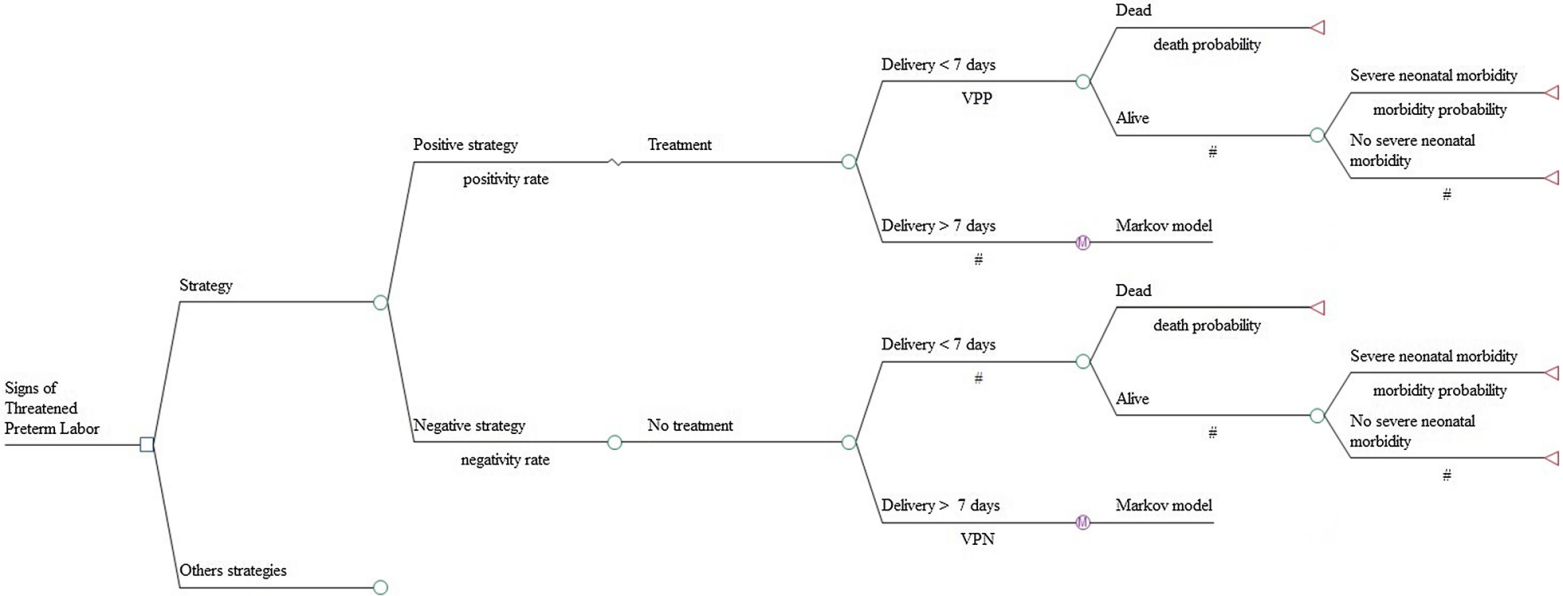
Decision analysis model. *vpp* positive predictive value. *vpn* negative predictive value

**Fig. 2:**
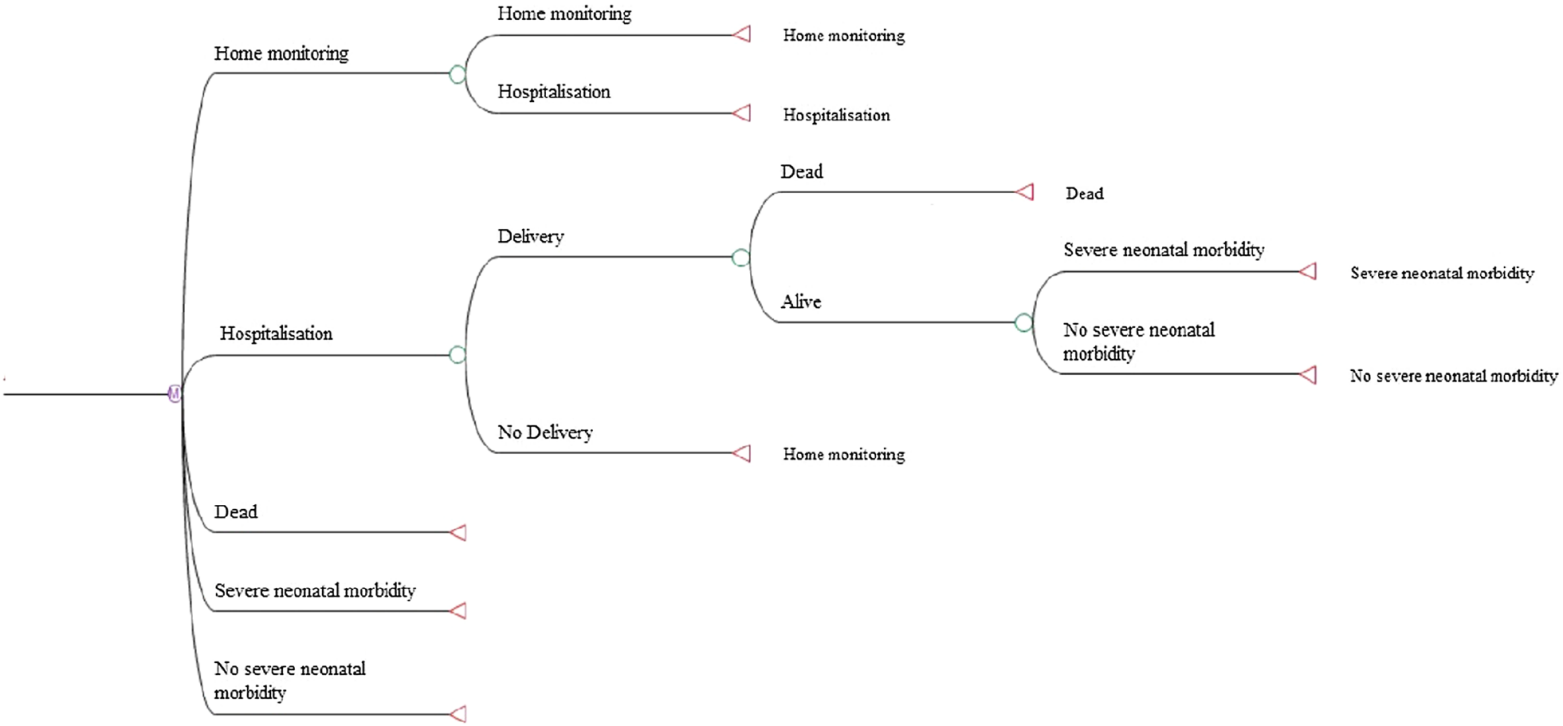
Markov model

For each compared strategy, in case of positive results corresponding to the probability of delivering within 7 days, women were hospitalized and treated. Treatment was defined as the administration of tocolytic agents and steroids, combined with the transfer of women to a perinatal center depending on the GA (Fig. 1). The possibility that a woman presented a positive result but did not deliver within the first 7 days was also modelled. In this case, a state-transition Markov model was used to simulate the probability of giving birth until 37 weeks gestation (Fig. 2). Four health states and one absorbing state were modelled: home monitoring, new hospitalisation, delivery with severe neonatal morbidity, delivery without severe neonatal morbidity and delivery with death of the new-born. At each new cycle of one week, the women could move from one state to another through predefined transition probabilities either until preterm delivery or until delivery at 37 weeks. A similar follow-up was modelled in case of negative test results.

### Model parameters

Two types of transition probabilities have to be distinguished: parameters which had to be estimated using data issued from the literature research, and those which were directly introduced into the model, based on national validated sources.

The parameters estimated from literature data concerned the probability of having a positive diagnostic test or not. It was estimated from a contingency table, based on an incidence of 9.7%, defined by the median of data issued from the literature and the sensitivity and specificity for each of the seven diagnostic tests (Table 1). It was therefore possible to estimate the probability of having a preterm delivery or not within 7 days in case of positive test (true and false positive situation respectively) and negative test (false and true negative situation) (Fig. 1).

**Table 1:** Values, ranges, distributions and references for parameters used in the decision tree

Variables	Value	Range^a^	Distribution	References
Incidence of preterm birth (%)	0.097	0.05–0.15	–	[8, 10, 13, 24, 25]
Diagnostic performances				
CL < 25 mm sensitivity	0.78	0.68–0.87	beta	[14]
CL < 25 mm specificity	0.71	0.67–0.74	beta	[14]
CL < 15 mm sensitivity	0.6	0.53–0.67	beta	[14]
CL < 15 mm specificity	0.9	0.89–0.92	beta	[14]
Qualitative fFN sensitivity	0.75	0.66–0.83	beta	[8, 20]
Qualitative fFN specificity	0.76	0.73–0.79	beta	[8, 20]
Quantitative fFN sensitivity	0.59	0.36–0.95	beta	[24]
Quantitative fFN specificity	0.94	0.91–0.97	beta	[24]
Cervical IL-6 sensitivity	0.83	0.53–1	beta	[10]
Cervical IL-6 specificity	0.94	0.89–0.99	beta	[10]
Combination CL < 18 mm, plasma RANTES, plasma IL-10 sensitivity	0.74	0.63–0.85	beta	[25]
			beta	
Combination CL < 18 mm, plasma RANTES, plasma IL-10 specificity	0.86	0.79–0.93	beta	[25]
			beta	
CL < 15 mm or CL 16–30 mm and qualitative fFN sensitivity	0.89	0.83–0.95	beta	[13]
CL < 15 mm or CL 16–30 mm and qualitative fFN specificity	0.7	0.67–0.73	beta	[13]
Probability of perinatal death				
If preterm birth [24–27] GA	0.392	0.35–0.44	beta	[29]
If preterm birth [28–31] GA	0.078	0.05–0.10	beta	[29]
If preterm birth [32–34] GA	0.033	0.009–0.057	beta	[29]
If preterm birth [24–34] GA	0.109	0.09–0.13	–	[29]
Probability of severe neonatal morbidity				[29]
If preterm birth [24–27] GA	0.303	0.25–0.36	beta	[29]
If preterm birth [28–31] GA	0.085	0.059–0.11	beta	[29]
If preterm birth [32–34] GA	0.016	0–0.034	beta	[29]
If preterm birth [24–34] GA	0.07	0.054–0.086	–	[29]
Probability of subsequent hospitalization				
If preterm birth [24–27] GA	0.13	–	–	PMSI
If preterm birth [28–31] GA	0.17	–	–	PMSI
If preterm birth [32–34] GA	0.27	–	–	PMSI
Probability of home follow-up				
If preterm birth [24–27] GA	0.94	–	–	PMSI
If preterm birth [28–31] GA	0.938	–	–	PMSI
If preterm birth [32–34] GA	0.916	–	–	PMSI

*GA* gestational age, *CL* cervical length, *fFN* fetal fibronectin, *IL* interleukin, *PMSI* Programme de medicalization du système d’information

^a^ Range used for univariate sensitivity analyses

Data directly based on national validated sources included:

- The probability of serious neonatal adverse events issued from the results of the EPIPAGE-2, a national cohort [26]. These events were defined as perinatal death or severe neonatal morbidity (severe bronchopulmonary dysplasia, severe necrotizing enterocolitis, severe retinopathy of prematurity, severe cerebral abnormalities on cranial ultrasonography).
- The probability of subsequent hospitalizations after discharge. It was issued from the data collected by a national medico-administrative database, the PMSI (Programme de médicalisation du système d’information). This database is used to determine the activity-based payment for hospitals in France. The reliability and validity of the PMSI data have already been assessed [27].

The parameters used in the model, the ranges over which they were tested and their sources are shown in Table 1.

### Analysis

#### Effectiveness

The effectiveness endpoint was the number of serious adverse events concerning the new-born, including either perinatal death or severe neonatal morbidity. We considered a score of 1 for death or severe neonatal morbidity and 0 otherwise.

#### Costs

The economic analysis was performed from the French healthcare system perspective. Only direct medical costs were taken into account. Costs were expressed in Euros (€) for the year 2012. Costs were not updated given the stability of prices in France (average annual variation of the consumer price index less than 1% between 2012 and 2017) [28].

The mothers’ and the newborns’ hospitalization was identified using their associated Diagnosis Related Groups (DRG). Their costs were estimated using the National cost survey sample named ‘Echelle nationale des coûts’ (ENC) [29]. The ENC estimates production costs of hospitalisation from a sample of public and private care centers. They were categorized in medical cost (consumable, diagnostic test, drugs, human resources) and structure cost (laundry, restauration, global logistics, depression and maintenance). Home follow-up care costs were estimated from the reimbursement of the national insurance health system concerning midwife consultation. All economic data are presented in Table 2.

**Table 2:** Values, ranges, distributions and references for economic parameters used in the decision tree (costs 2012, €)

	Value	Range	Distribution	References
Weekly home follow-up	42	–	–	NIHS
[24–27] GA				
Prenatal hospitalization for TPL	1445	532–2736	Gamma	NCSS
Preterm labor	2877	1327–11,570	Log-normal	NCSS
Preterm labor hospitalization	2890	1332–11,594	Log-normal	NCSS
Perinatal death	1867	323–80,068	Log-normal	NCSS
Neonatal hospitalization without morbidity	27,295	1380–78,887	Log-normal	NCSS
Neonatal hospitalization with morbidity	58,775	2406–79,591	Gamma	NCSS
[28–31] GA				
Prenatal hospitalization for TPL	1762	532–5370	Gamma	NCSS
Preterm labor	3606	1318–11,525	Log-normal	NCSS
Preterm labor hospitalization	3639	1328–11,626	Log-normal	NCSS
Perinatal death	1235	323–18,976	Log-normal	NCSS
Neonatal hospitalization without morbidity	19,748	1380–79,394	Log-normal	NCSS
Neonatal hospitalization with morbidity	40,419	2150–79,690	Gamma	NCSS
[32–34] GA				
Prenatal hospitalization for TPL	2113	532–11,625	Gamma	NCSS
Preterm labor	4482	1334–11,625	Log-normal	NCSS
Preterm labor hospitalization	4517	1343–11,821	Log-normal	NCSS
Perinatal death	1606	323–57,387	Log-normal	NCSS
Neonatal hospitalization without morbidity	10,907	1380–61,756	Log-normal	NCSS
Neonatal hospitalization with morbidity	20,331	1380–36,321	Gamma	NCSS

*GA* gestational age, *TPL* threatened preterm labor, *NIHS* National Insurance Health System, *NCSS* National Cost Survey Sample

#### Cost-effectiveness analysis

All strategies were compared with each other. Strategies were ranked from the least to the most costly. Strategies that were more costly and less effective (i.e. presenting a higher number of serious adverse events) than the next alternative were excluded by simple dominance. Strategies presenting a higher incremental cost-effectiveness ratio (ICER) than that of the next most effective alternative were excluded by extended dominance. ICER was calculated according to the following formula:

$${\text{ICER}} = \left( {{\text{Mean Cost}}_{\text{test n}} - {\text{Mean Cost}}_{{{\text{test n}} - 1}} } \right)/\left( {{\text{Mean effectiveness}}_{\text{test n}} - {\text{Mean effectiveness}}_{{{\text{test n}} - 1}} } \right)$$

Four cost-effectiveness analyses were performed: one for each GA group (24–27, 28–31 and 32–34 weeks), and one for the whole period of 24–34 weeks GA, which was obtained by adjusting the cost and effectiveness results of each GA group by the proportion of mothers in each of these periods. As the time frame was less than one year, costs and effectiveness were not discounted.

#### Sensitivity analyses

Three deterministic sensitivity analyses were performed to test the robustness of the model. The first analysis concerned the incidence of preterm birth which was fixed at 5% and then at 15%.

The second analysis concerned the diagnostic performances of the tests. The maximum values of sensitivities and specificities were first simultaneously tested, and a similar analysis was then performed with their minimum values.

#### Probabilistic analyses

A Monte Carlo simulation was also performed. The Monte Carlo analysis draws a cost-effectiveness plane divided into four quadrants [30]: the northeast (NE) quadrant contained situations where incremental costs and effects were both positive (ΔE > 0 and ΔC > 0), indicating that the new test was dominated by the alternative test (because in our study, a high level of effectiveness corresponded to a high level of severe neonatal events for the newborn). The southwest (SW) quadrant contained the opposite situation where the new test dominated the alternative (ΔE < 0 and ΔC < 0). The northwest (NW) quadrant corresponded to the situation where incremental costs were positive and incremental effects negative (ΔE < 0 and ΔC > 0), indicating that a trade-off needed to be made and an ICER had to be calculated. Finally, in the southeast (SE) quadrant, we find a situation with negative incremental effects as well as cost savings (ΔE > 0 and ΔC < 0) [31]. The distribution of transition probabilities and costs were sampled in 5000 consecutive iterations (Table 1).

## Results

### Baseline cost-effectiveness analysis

Results showed that at 24–34 GA, cervical length < 15 mm, or a positive qualitative fetal fibronectin test when CL was between 16 and 30 mm (S_7_) was the least costly and the most effective strategy (because it is associated with the lowest number of neonatal serious adverse events) and dominated the reference strategy (CL < 25 mm) and all other alternatives (Table 3). The rate of perinatal death or severe neonatal morbidity was decreased in a range varying between 9 and 15% (from 2.33 to 4.2 serious neonatal adverse events avoided per 1000 new-borns) and cost saving of between 25 and 31% (from €1107 to €1481 per mother–child) depending on the strategies compared.

**Table 3:** Cost and effectiveness of seven diagnostic strategies for threatened preterm labor at 24–34 weeks gestational age

Strategies in order of decreasing cost effectiveness	Cost per mother–child, €	Neonatal serious adverse events	ICER^a^, €
S_7_: CL < 15 mm or CL [16–30 mm] and qualitative fFN	3237	0.0233	–
S_3_: Quantitative fFN	4344	0.0256	481,304 (dominated)
S_2_: Qualitative fFN	4385	0.0260	425,034 (dominated)
S_ref_: CL < 25 mm	4400	0.0262	401,034 (dominated)
S_4_: IL-6 cervical	4415	0.0264	380,000 (dominated)
S_5_: Combination CL < 18 mm, RANTES plasma, IL-10 plasma	4431	0.0266	361,818 (dominated)
S_6_: CL < 15 mm	4718	0.0275	352,619 (dominated)

*CL* cervical length, *fFN* fetal fibronectin, *IL* interleukin

^a^ Incremental cost-effectiveness ratio expressed in terms of cost per additional serious adverse event. For example, the ICER 481,304 € should be interpreted as following: S_3_ is associated to an added cost of 481,304 € per additional neonatal adverse event compared to S_7_

Similar results were found for each gestational age group (24–27; 28–31; 32–34). Results also showed that the earlier the prematurity, the higher the number of avoided serious adverse neonatal events when strategies were compared (Additional file 1: Supplement A).

### Deterministic sensitivity analyses

Results issued from the deterministic analyses concerning the incidence of preterm birth confirmed the efficiency of the association of CL and qualitative fFN (S_7_) compared with the other strategies. Results issued from the analyses using the minimum and the maximum values of the diagnostic test performances also confirmed this result (Table 4).

**Table 4:** Deterministic sensitivity analyses

	Cost per mother–child, €	Neonatal serious adverse events	ICER^a^, €
Minimum values of sensitivity and maximum values of specificity			
S_7_: CL < 15 mm or CL [16–30 mm] and qualitative fFN	3040	0.0219	–
S_2_: Qualitative fFN	4359	0.0258	338,205
S_5_: CL < 18 mm, plasma RANTES and plasma IL-10	4382	0.0260	327,317
S_ref_: CL < 25 mm	4388	0.0261	320,952
S_4_: Cervical IL6	4465	0.0269	285,000
S_3_: Quantitative fFN	4492	0.0269	290,400
S_6_: CL < 15 mm	4592	0.0264	344,889
Maximum values of sensitivity and minimum values of specificity			
S_7_: CL < 15 mm or CL [16–30 mm] and qualitative fFN	3300	0.0236	–
S_2_: Qualitative fFN	4381	0.0260	450,417
S_5_: CL < 18 mm, plasma RANTES and plasma IL-10	4414	0.0264	397,857
S_ref_: CL < 25 mm	4427	0.0265	388,621
S_4_: Cervical IL-6	4465	0.0269	353,030
S_3_: Quantitative fFN	4473	0.0270	345,000
S_6_: CL < 15 mm	4653	0.0268	422,813
Incidence of preterm birth of 5%			
S_7_: CL < 15 mm or CL [16–30 mm] and qualitative fFN	1927	0.0119	–
S_3_: Quantitative fFN	3517	0.0168	324,490
S_ref_: CL < 25 mm	3560	0.0172	308,113
S_4_: Cervical IL-6	3560	0.0172	308,113
S_2_: Qualitative fFN	3587	0.0175	296,429
S_5_: CL < 18 mm, plasma RANTES and plasma IL-10	3604	0.0177	289,138
S_6_: CL < 15 mm	3719	0.0143	746,667
Incidence of preterm birth of 15%			
S_7_: CL < 15 mm or CL [16–30 mm] and qualitative fFN	4121	0.0337	–
S_ref_: CL < 25 mm	5414	0.037	391,818
S_3_: Quantitative fFN	5437	0.0373	365,556
S_5_: CL < 18 mm, plasma RANTES and plasma IL-10	5443	0.0374	357,297
S_2_: Qualitative fFN	5452	0.0374	359,730
S_4_: Cervical IL-6	5514	0.0381	316,591
S_6_: CL < 15 mm	5636	0.0295	− 360,714

*CL* cervical length, *fFN* fetal fibronectin, *IL* interleukin

^a^ Incremental cost-effectiveness ratio expressed in terms of cost per additional serious adverse event compared to S_7_. A positive ICERs correspond to an added cost per additional neonatal adverse event compared to S_7_. All strategies with a positive ICER are dominated by S_7_. The negative ICER corresponds to added costs to avoid one additional serious adverse event compared to S_7_

### Probabilistic analysis

Table 5 indicates the proportion of points for each GA, representing pairs of incremental costs and effectiveness in each of these four quadrants. These points were issued from the comparison between the most efficient strategy, identified with the baseline cost-effectiveness analysis of this work, and the six other diagnostic tests.

**Table 5:** Probabilistic analysis (5000 iterations): proportions (%) of pairs of incremental cost and incremental severe adverse neonatal events associated with CL < 15 mm or CL [16–30 mm] and fFN qualitative compared to each of the six other strategies

	∆E < 0 and ∆C < 0^a^	∆E < 0 and ∆C > 0^b^	∆E > 0 and ∆C < 0^c^	∆E > 0 and ∆C > 0^d^
Strategies [24–27] GA				
S_6_: CL < 15 mm	90	1	0	9
S_ref_: CL < 25 mm	24	47	0	29
S_2_: Qualitative fFN	20	47	0	33
S_3_: Quantitative fFN	26	23	0	51
S_4_: Cervical IL-6	41	18	0	41
S_5_: CL < 18 mm, plasma RANTES and plasma IL-10	27	24	0	49
Strategies [28–31] GA				
S_6_: CL < 15 mm	92	0	3	5
S_ref_: CL < 25 mm	28	45	0	27
S_2_: Qualitative fFN	24	45	0	31
S_3_: Quantitative fFN	28	20	0	52
S_4_: Cervical IL-6	44	17	0	39
S_5_: CL < 18 mm, plasma RANTES and plasma IL-10	28	23	0	49
Strategies [32–34] GA				
S_6_: CL < 15 mm	96	0	3	1
S_ref_: CL < 25 mm	59	25	3	13
S_2_: Qualitative fFN	54	26	3	17
S_3_: Quantitative fFN	43	12	5	41
S_4_: Cervical IL-6	55	10	3	31
S_5_: CL < 18 mm, plasma RANTES and plasma IL-10	45	14	5	36

*GA* gestational age, *CL* cervical length, *fFN* fetal fibronectin, *IL* interleukin

^a^ The southwest quadrant (SW) represented the proportion where CL < 15 mm or CL [16–30 mm] and fFN qualitative dominated the alternative strategies

^b^ The northwest quadrant (NW) represented the proportion where CL < 15 mm or CL [16–30 mm] and fFN qualitative was more effective but more costly

^c^ The southeast quadrant (SE) represented the proportion where CL < 15 mm or CL [16–30 mm] and fFN qualitative was less effective and less costly

^d^ The northeast quadrant (NE) represented the proportion where CL < 15 mm or CL [16–30 mm] and fFN qualitative was less effective and more costly

The results showed that when the association of CL and qualitative fFN was compared with CL < 15 mm (S_6_), most of the pairs of incremental costs and effectiveness were contained in the SW quadrant of the cost-effectiveness plane (ΔE < 0 and ΔC < 0, i.e. showing a higher effectiveness and cost-savings associated with CL and qualitative fFN): at 24–27 GA, the probability that the association of CL and qualitative fFN dominates CL < 15 mm was estimated to be 90%; at 28–31 and 32–34 GA, the analysis depicted a probability of 92 and 96% respectively.

More uncertainty was observed concerning the comparison between CL and qualitative fFN and the five other strategies (S_ref_, S_2_, S_3_, S_4_ and S_5_): at 24–27 GA, the proportion of pairs of incremental results was split between the SW quadrant and the NW quadrant range between 49 and 71% according to the strategies. At 28–31, this proportion varied between 48 and 73%. At 32–34, the range was 55–84%. Moreover, the association of CL and qualitative fFN (S_7_) was dominated (NE quadrant) by S_3_, S_4_, S_5_ in almost half of the cases and whatever the GA (Table 5 and Additional file 2: Supplement B).

## Discussion

### Summary of key findings

The results showed that among seven diagnostic strategies in singleton pregnancy presenting TPL between the GA of 24 and 34 weeks, CL less than 15 mm or a positive qualitative fFN when CL was between 16 and 30 mm (S_7_), was the most efficient diagnostic strategy and led to a reduction in neonatal morbidity and mortality and significant cost savings compared to all other alternative strategies (with a cost savings of 1481€ per mother–child and 4.2 serious neonatal adverse events avoided per 1000 new-borns). The deterministic and probabilistic analyses confirmed the domination of the association of CL and qualitative fFN over the other tests and especially over CL < 15 mm which was the least effective and the most costly strategy. This can be explained by the fact that this strategy had a poor sensitivity compared to the combination of strategies.

### Comparison with other studies

To the best of our knowledge, relatively few cost-effectiveness analyses on this topic have been performed. Most of the studies did not include the combination of CL and qualitative fFN [20–23], except the study conducted by van Baaren et al. in the Netherlands who found that testing fFN in women with CL between 10 and 30 mm was the most efficient strategy [19]. Both our decision analysis models provided arguments in favor of this strategy for the international medical community [15].

The previous observational studies showed that CL combined with fFN could improve the identification of women with a low risk of delivering spontaneously within 7 days [8, 32–34] and thus reduce costs and the number of hospitalizations [21]. The clinical trial conducted by Ness et al. also showed that CL combined with fFN was also associated with reduced evaluation time in triage for women with CL ≥ 30 mm [36]. In consequence, this strategy (CL less than 15 mm or a positive qualitative fFN when CL was between 16 and 30 mm (S_7_)) could be easily applied in current obstetrics practice whatever the type of center. However, it requires the application of the standardized protocol described by Schmitz et al. [35], as clinicians must sample qualitative fFN and then measure CL by transvaginal ultrasound before making a decision.

### Strengths and limitations

The main strength of our study is that it was based on reliable data from three official and validated sources: the Epipage 2-cohort which provided neonatal morbidity and mortality data, PMSI which is a national medico-administrative database, and the National cost sample survey which provided costs issued from public and private care centers [29]. These data gave us the opportunity to provide detailed results according to GA. The stratified cost-effectiveness analysis showed that the strategy combining CL with qualitative fFN had a positive economic and medical impact according to GA groups: at 24–27 weeks GA, the number of serious adverse neonatal events was much higher compared to 28–31 and 32–34 GA with an overall cost not exceeding €1500 per mother child. This overall cost should be traded-off with the cost of complications associated with high prematurity [36, 37] and the cost of follow-up for these children over a longer period of time. The national sources of data also provided enough robust parameters to be able to implement a Markov model in our decision tree to take into account the complexity of mother–child management and to avoid underestimating the costs associated with subsequent hospitalization after discharge. Another strength was the selection for which, contrary to the study conducted by van Baaren, only the studies including the same criteria of inclusion population were included in our analyses, therefore limiting the selection bias.

Our analysis does present some limits. Firstly, our population did not include all preterm births because women presenting either a disease associated with a high risk of preterm birth or multiple pregnancy were excluded from our analyses because these medical situations do not require the use of diagnostic tests for TPL. Moreover, the data on diagnostic performance used for our study was all derived from observational studies, which can be prone to bias. Then the choice of the reference strategy in our baseline cost-effectiveness analysis. Currently, no diagnostic strategy is clearly recommended by international guidelines, and our choice to have taken cervical length as the reference strategy may be controversial. However, this choice had no effect on our results because all the other strategies were dominated.

Another limit concerns the strategies modelled: even if we modelled the use of new diagnostic tests such as quantitative fetal fibronectin or proteins in maternal serum, the combination of quantitative fFN and CL was not included in the model due to a lack of data.

For the economic analysis, only direct medical costs in public hospitals were taken into account, which raises the question of the transferability of the results to other countries where the organization and the financing of care is different. We also made the choice not to perform a cost-utility analysis because the main goal of our work was to assess the clinical consequences associated with threatened preterm labor. Prematurity is associated with long-term neuro-motor, sensory and cognitive disabilities. Given the economic consequences linked to these impairments, an analysis using Quality Adjusted Life Year (QALY) would have been relevant. Unfortunately, the medical and economic data required for this type of analysis were not available and would have required conducting further studies.

## Conclusion

The strategy that combines CL with qualitative fFN, defined as positive if either CL < 15 mm or if CL is 16–30 mm and qualitative fFN is positive, appeared to be the most efficient strategy. Our findings could lead to a significant reduction of medical costs. Furthermore, this decision analysis provides arguments for establishing new guidelines, and informing the daily practice of clinicians in regional perinatal networks. Indeed, our suggested strategy is based on current obstetric practices, and represents no additional costs compared to the most used diagnostic test at the moment in France. This test can be implemented whatever the level of maternity center, which would make it easier to move women at high risk of delivery towards a center equipped to optimize the health of premature newborns.

## Additional files

**Additional file 1: Supplement A.** Costs and effectiveness outcomes of seven diagnostic strategies for threatened preterm labor according to the gestational age.

**Additional file 2: Supplement B.** Scatter plots showing proportions (%) of pairs of incremental cost and incremental severe adverse neonatal events associated with CL < 15 mm or CL [16–30 mm] and fFN qualitative compared to strategy 3 and 6 according to GA.

## Abbreviations

CL: cervical length
DRG: diagnoses relate groups
ENC: echelle nationale des coûts
fFN: fetal fibronectin
GA: gestational age
ICER: incremental cost-effectiveness ratio
IL-6: interleukin-6
NE: northeast
NW: northwest
PTD: preterm delivery
PMSI: programme de médicalisation des systèmes d’information
RANTES: plasma on activation normal T-expressed and secreted regulated
SE: southeast
SW: southwest
TPL: threatened preterm labor

## References

[1] Blencowe H, Cousens S, Oestergaard MZ, Chou D, Moller AB, Narwal R (2012). National, regional, and worldwide estimates of preterm birth rates in the year 2010 with time trends since 1990 for selected countries: a systematic analysis and implications. Lancet.

[2] Goldenberg RL, Culhane JF, Iams JD, Romero R (2008). Epidemiology and causes of preterm birth. Lancet.

[3] Nicholson WK, Frick KD, Powe NR (2000). Economic burden of hospitalizations for preterm labor in the United States. Obstet Gynecol.

[4] Sagot P, Roze C, Rigal E, Dantal F, De Morel P, Samake M (1990). Birth before 33 weeks gestational age. The significance of in utero-to-birth transfer in the Department of Perinatology. Rev Fr Gynecol Obstet.

[5] Sanchez-Ramos L, Delke I, Zamora J, Kaunitz AM (2009). Fetal fibronectin as a short-term predictor of preterm birth in symptomatic patients: a meta-analysis. Obstet Gynecol.

[6] McPheeters ML, Miller WC, Hartmann KE, Savitz DA, Kaufman JS, Garrett JM (2005). The epidemiology of threatened preterm labor: a prospective cohort study. Am J Obstet Gynecol.

[7] Sosa CG, Althabe F, Belizan JM, Bergel E (2015). Bed rest in singleton pregnancies for preventing preterm birth. Cochrane Database Syst Rev.

[8] DeFranco EA, Lewis DF, Odibo AO (2013). Improving the screening accuracy for preterm labor: is the combination of fetal fibronectin and cervical length in symptomatic patients a useful predictor of preterm birth? A systematic review. Am J Obstet Gynecol.

[9] Boots AB, Sanchez-Ramos L, Bowers DM, Kaunitz AM, Zamora J, Schlattmann P (2014). The short-term prediction of preterm birth: a systematic review and diagnostic metaanalysis. Am J Obstet Gynecol.

[10] Brik M, Antonio P, Perales-Puchalt A, Diago V, Perales A (2011). Cervical interleukin-6 as a predictive test for preterm delivery in symptomatic women: preliminary results. Eur J Obstet Gynecol Reprod Biol.

[11] Goldenberg RL, Iams JD, Das A, Mercer BM, Meis PJ, Moawad AH (2000). The Preterm prediction study: sequential cervical length and fetal fibronectin testing for the prediction of spontaneous preterm birth. National Institute of Child Health and Human Development Maternal-Fetal Medicine Units Network. Am J Obstet Gynecol.

[12] Iams JD, Goldenberg RL, Meis PJ, Mercer BM, Moawad A, Das A (1996). The length of the cervix and the risk of spontaneous premature delivery. National Institute of Child Health and Human Development Maternal Fetal Medicine Unit Network. N Engl J Med.

[13] Schmitz T, Kayem G, Maillard F, Lebret MT, Cabrol D, Goffinet F (2008). Selective use of sonographic cervical length measurement for predicting imminent preterm delivery in women with preterm labor and intact membranes. Ultrasound Obstet Gynecol.

[14] Sotiriadis A, Papatheodorou S, Kavvadias A, Makrydimas G (2010). Transvaginal cervical length measurement for prediction of preterm birth in women with threatened preterm labor: a meta-analysis. Ultrasound Obstet Gynecol.

[15] Guidelines RCOG: Tocolysis for Women in Preterm Labour. Green–top Guideline February 2011, No. 1b.

[16] American College of Obstetrcian and Gynecologists (2012). Committee on Practice B-O: ACOG practice bulletin no. 127: Management of preterm labor. Obstet Gynecol.

[17] College National Gynécologie Obstétrique Français (2002). La menace d’accouchement prématuré à membranes intactes. J Gynecol Obstet Biol Reprod.

[18] Sentilhes L, Senat MV, Ancel PY, Azria E, Benoist G, Blanc J (2016). Prevention of spontaneous preterm birth (excluding preterm premature rupture of membranes): guidelines for clinical practice—text of the guidelines (short text). J Gynecol Obstet Biol Reprod (Paris).

[19] Van Baaren GJ, Vis JY, Grobman WA, Bossuyt PM, Opmeer BC, Mol BW (2013). Cost-effectiveness analysis of cervical length measurement and fibronectin testing in women with threatened preterm labor. Am J Obstet Gynecol.

[20] Honest H, Forbes CA, Duree KH, Norman G, Duffy SB, Tsourapas A (2009). Screening to prevent spontaneous preterm birth: systematic reviews of accuracy and effectiveness literature with economic modelling. Health Technol Assess.

[21] Rose CH, McWeeney DT, Brost BC, Davies NP, Watson WJ (2010). Cost-effective standardization of preterm labor evaluation. Am J Obstet Gynecol.

[22] Deshpande SN, van Asselt AD, Tomini F, Armstrong N, Allen A, Noake C (2013). Rapid fetal fibronectin testing to predict preterm birth in women with symptoms of premature labour: a systematic review and cost analysis. Health Technol Assess.

[23] Mozurkewich EL, Naglie G, Krahn MD, Hayashi RH (2000). Predicting preterm birth: a cost-effectiveness analysis. Am J Obstet Gynecol.

[24] Abbott DS, Radford SK, Seed PT, Tribe RM, Shennan AH (2013). Evaluation of a quantitative fetal fibronectin test for spontaneous preterm birth in symptomatic women. Am J Obstet Gynecol.

[25] Tsiartas P, Holst RM, Wennerholm UB, Hagberg H, Hougaard DM, Skogstrand K (2012). Prediction of spontaneous preterm delivery in women with threatened preterm labour: a prospective cohort study of multiple proteins in maternal serum. BJOG.

[26] Ancel PY, Goffinet F, Group E-W, Kuhn P, Langer B, Matis J (2015). Survival and morbidity of preterm children born at 22 through 34 weeks’ gestation in France in 2011: results of the EPIPAGE-2 cohort study. JAMA Pediatr.

[27] Quantin C, Benzenine E, Ferdynus C, Sediki M, Auverlot B, Abrahamowicz M (2013). Advantages and limitations of using national administrative data on obstetric blood transfusions to estimate the frequency of obstetric hemorrhages. J Public Health (Oxf).

[28] Institut National de la Statistique et des Etudes Economiques. https://www.insee.fr/fr/statistiques/2122401. Accessed 01 june 2017.

[29] Agence Technique de l’Information sur l’Hospitalisation (ATIH). http://www.atih.sante.fr/enc-mco-donnees-2012. Accessed 01 june 2016.

[30] Black WC (1990). The CE plane: a graphic representation of cost-effectiveness. Med Decis Making.

[31] Al MJ (2013). Cost-effectiveness acceptability curves revisited. Pharmacoeconomics.

[32] Soilly AL, Lejeune C, Quantin C, Bejean S, Gouyon JB (2014). Economic analysis of the costs associated with prematurity from a literature review. Public Health.

[33] Van Baaren GJ, Peelen MJ, Schuit E, van der Post JA, Mol BW, Kok M (2015). Preterm birth in singleton and multiple pregnancies: evaluation of costs and perinatal outcomes. Eur J Obstet Gynecol Reprod Biol.

[34] Van Baaren GJ, Vis JY, Wilms FF, Oudijk MA, Kwee A, Porath MM (2014). Predictive value of cervical length measurement and fibronectin testing in threatened preterm labor. Obstet Gynecol.

[35] Deplagne C, Maurice-Tison S, Coatleven F, Vandenbossche F, Horovitz J (2010). Sequential use of cervical length measurement before fetal fibronectin detection to predict spontaneous preterm delivery in women with preterm labor. J Gynecol Obstet Biol Reprod (Paris).

[36] Ness A, Visintine J, Ricci E, Berghella V (2007). Does knowledge of cervical length and fetal fibronectin affect management of women with threatened preterm labor? A randomized trial. Am J Obstet Gynecol.

[37] Schmitz T, Maillard F, Bessard-Bacquaert S, Kayem G, Fulla Y, Cabrol D (2006). Selective use of fetal fibronectin detection after cervical length measurement to predict spontaneous preterm delivery in women with preterm labor. Am J Obstet Gynecol.

