# Supplementary material for: Cost-effectiveness of diagnostic tests for threatened preterm labor in singleton pregnancy in France
Source: Cost Eff Resour Alloc. 2018 Jun 14;16:21. doi: 10.1186/s12962-018-0106-y (PMC6003030; doi:10.1186/s12962-018-0106-y)
Supplement: Supplementary file 1 — Additional file 1: Supplement A. Costs and effectiveness outcomes of seven diagnostic strategies for threatened preterm labor according to the gestational age. [file 12962_2018_106_MOESM1_ESM.docx]

**Additional files. Supplement A. Costs and effectiveness outcomes of seven diagnostic strategies for threatened preterm labor according to the gestational age**

| **Gestational Age** | **24-27** | | | **28-31** | | | **32-34** | | |
| --- | --- | --- | --- | --- | --- | --- | --- | --- | --- |
| **Strategies** | **Cost per  mother-child, €** | **Neonatal serious adverse events** | **ICER^a^** | **Cost per  mother-child, €** | **Neonatal serious adverse events** | **ICER^a^** | **Cost per  mother-child, €** | **Neonatal serious adverse events** | **ICER^a^** |
| S_7_: CL< 15 mm or CL [16-30mm] and  fFN qualitative | 3938 | 0.0628 | - | 3566 | 0.0170 | - | 2720 | 0.0055 | - |
| S_3_: Quantitative fFN | 5142 | 0.0684 | 215,000 | 4715 | 0.0187 | 675,882 | 3757 | 0.0063 | 1,296,250 |
| S_2_: Qualitative fFN | 5194 | 0.0696 | 184,706 | 4762 | 0.0190 | 598,000 | 3789 | 0.0064 | 1,187,778 |
| S_ref_: CL<25mm | 5214 | 0.0700 | 177,222 | 4780 | 0.0191 | 578,095 | 3801 | 0.0065 | 1,081,000 |
| S_4_: Cervical IL-6 | 5233 | 0.0705 | 168,182 | 4799 | 0.0192 | 560,455 | 3813 | 0.0065 | 1,093,000 |
| S_5_: CL <18mm, plasma RANTES and plasma IL-10 | 5243 | 0.0711 | 157,229 | 4821 | 0.0194 | 522,917 | 3827 | 0.0066 | 1,006,364 |
| S_6_: CL <15mm | 5487 | 0.0736 | 508,056 | 5074 | 0.0201 | 486,452 | 4153 | 0.0069 | 1,023,571 |

GA: Gestational Age. CL: Cervical length. fFN: fetal fibronectin. IL: Interleukin.

^a^ Incremental cost-effectiveness ratio expressed in terms of cost per additional serious adverse event compared to S_7_. A positive ICERs correspond to added cost per additional neonatal adverse event compared to S7. All strategies with a positive ICER are dominated by S7
