# Supplementary material for: Cost-effectiveness of diagnostic tests for threatened preterm labor in singleton pregnancy in France
Source: Cost Eff Resour Alloc. 2018 Jun 14;16:21. doi: 10.1186/s12962-018-0106-y (PMC6003030; doi:10.1186/s12962-018-0106-y)
Supplement: Supplementary file 2 — Additional file 2: Supplement B. Scatter plots showing proportions (%) of pairs of incremental cost and incremental severe adverse neonatal events associated with CL < 15 mm or CL [16–30 mm] and fFN qualitative compared to strategy 3 and 6 according to GA. [file 12962_2018_106_MOESM2_ESM.docx]

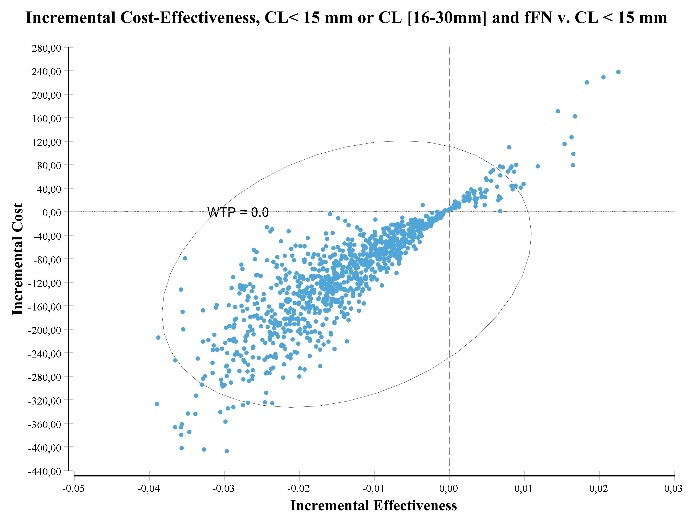


**A: Scatter plot comparing S_7_ to S_6_ at 24- 27GA.**

**
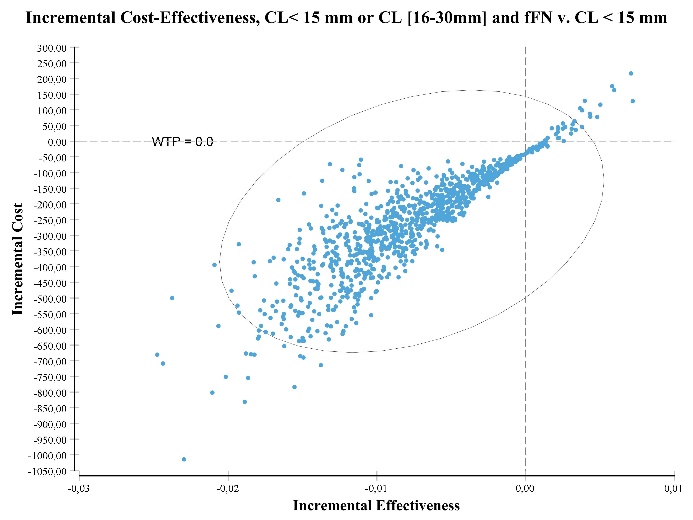
**

**B: Scatter plot comparing S_7_ to S_6_ at 28- 31GA.**

**
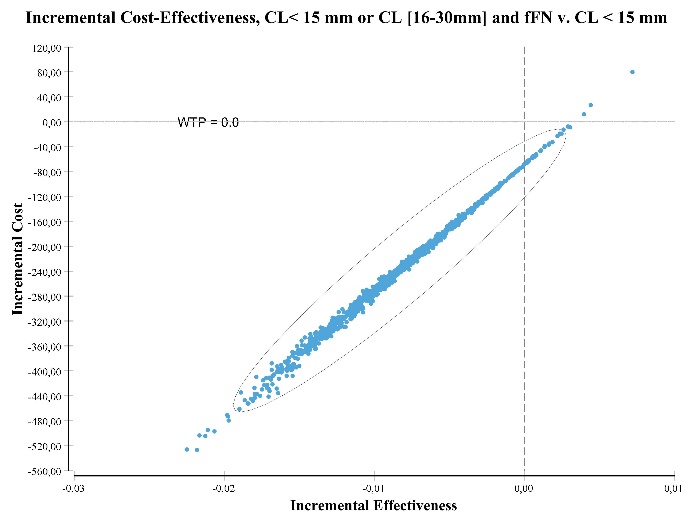
**

**C: Scatter plot comparing S_7_ to S_6_ at 32- 34 GA.**

**Additional files. Supplement B. Scatter plots showing proportions (%) of pairs of incremental cost and incremental severe adverse neonatal events associated with CL< 15 mm or CL [16-30mm] and fFN qualitative compared to strategy 3 and 6 according to GA.**


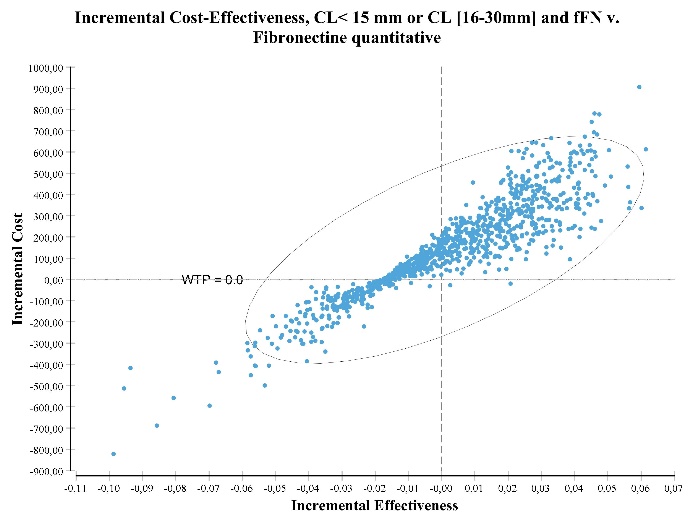


**D: Scatter plot comparing S_7_ to S_3_ at 24- 27GA.**


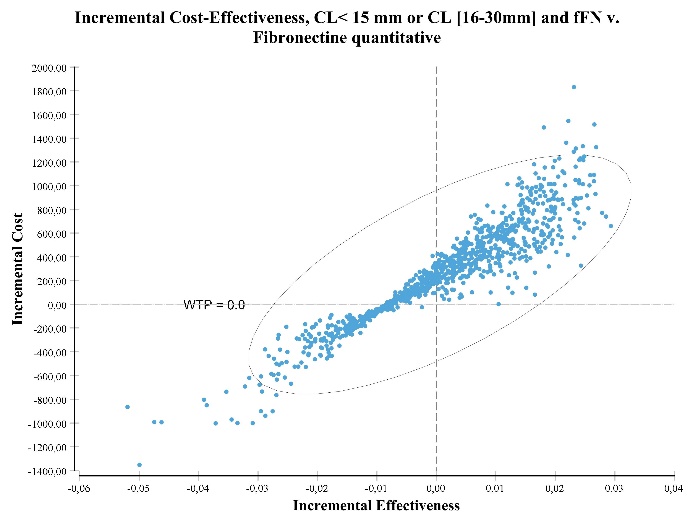


**E: Scatter plot comparing S_7_ to S_3_ at 28- 31GA.**

**
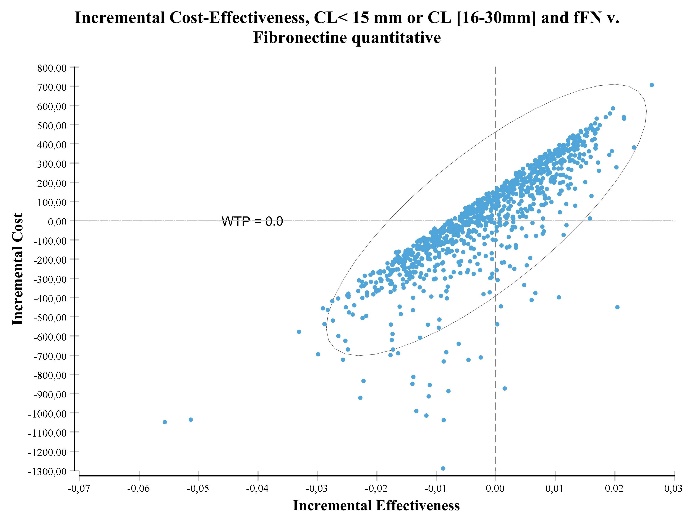
**

**F: Scatter plot comparing S_7_ to S_3_ at 32- 34GA.**
